# Endoscopic Third Ventriculostomy and Cortical Biopsy in Patients With Normal Pressure Hydrocephalus

**DOI:** 10.7759/cureus.31523

**Published:** 2022-11-15

**Authors:** George W Koutsouras, Emma Steinmetz, Michael Tichenor, Brianna Schmidt, YS Mohan, Satish Krishnamurthy

**Affiliations:** 1 Neurosurgery, State University of New York Upstate Medical University, Syracuse, USA; 2 Surgery, University of South Carolina, Columbia, USA; 3 Neurosurgery, Henry Ford Health System, Detroit, USA

**Keywords:** dementia, neuritic plaques, neuropathology, endoscopic third ventriculostomy, normal pressure hydrocephalus

## Abstract

Introduction

Normal pressure hydrocephalus (NPH) has conventionally been treated by placement of a ventriculoperitoneal shunt. However, it can also be treated with a less invasive technique, an endoscopic third ventriculostomy (ETV). Unfortunately, there is a lack of evidence on the characteristics of NPH patients who are most likely to benefit from ETV. This study seeks to identify if patients at risk of dementia with NPH should be candidates for an ETV.

Methodology

Thirty-six NPH patients who underwent ETV at two institutions between July 2007 and December 2014 were pre-surgically assessed for various risk factors. At the time of ETV, a cortical biopsy was obtained and assessed for plaques consistent with dementia. Post-procedure, patients were followed and assessed for symptoms such as gait improvement, headache, memory problems, incontinence, and dementia. ETV success was defined as an improvement in gait.

Results

The mean age of patients with successful ETVs was 65.8 ± 6.0 versus 74.5 ± 7.0 for failed ETVs. Sixty-seven percent of patients with negative biopsies showed gait improvement by the final follow-up appointment as compared to only 33% of patients with positive biopsies (p>0.05). Younger age was correlated with successful ETV (p=.003). Memory disturbance (p<0.05) and incontinence (p<0.05) after surgery were both associated with a lack of gait improvement at the final follow-up.

Conclusion

Biopsy was not a significant predictor of ETV success; however, there was a correlation between younger age and ETV success. Additional studies are required to determine if there is a relationship between cortical biopsy findings and ETV success.

## Introduction

Hydrocephalus is a CNS disorder characterized by abnormal accumulation of CSF in the cerebral ventricles, causing dilation and associated brain tissue injury [1]. A subset of this condition, idiopathic normal pressure hydrocephalus (iNPH), is unique in that opening pressure is not completely normal upon lumbar puncture testing. Classic iNPH presents as a triad of gait disturbance, dementia, and urinary incontinence and is most commonly seen in the elderly [2]. These symptoms are not necessarily reversible and can become chronically disabling if the condition is not treated in a timely fashion. As such, it remains essential to continue to investigate ways to optimize the treatment for patients afflicted with this condition [3]. As patients with iNPH are typically older individuals, neurological pathologies may coexist. Studies have found that 40-75% of idiopathic iNPH patients have beta-amyloid plaques or other histology consistent with Alzheimer’s disease (AD). Patients with plaques consistent with AD or vascular dementia are less likely to benefit from shunting procedures than patients without these histological markers [4].

Traditionally, NPH has been treated using ventriculoperitoneal or ventriculoatrial shunts [5]. However, these procedures are not always successful, and they come along with a host of potential complications, such as bowel perforation, under or over-draining, and infection [6]. There is potential for the utility of endoscopic third ventriculostomy (ETV) as a surgical option, but the evidence is lacking on the utility of this approach [7]. Traditionally, endoscopic third ventriculostomy has been used to treat obstructive hydrocephalus. Studies have demonstrated improvements in both gait and cognitive dysfunction in patients with obstructive hydrocephalus, symptoms that are commonly seen in iNPH [8-10]. Varying surgical success rates have been described for iNPH, ranging from 30 to 90% [6]. It is unclear whether the coinciding feature of dementia, diagnosed with cortical biopsy at the time of surgery, contributes to the success rates in ETV for NPH. By better understanding the factors associated with clinical outcomes, surgeons can better advise their patients on whether the multiple benefits of an ETV outweigh the risk of failure. This study aimed to identify that those diagnosed with a neurodegenerative disease such as AD on cortical biopsy are most likely to experience success or failure of an ETV procedure.

## Materials and methods

We retrospectively analyzed patients diagnosed with NPH using diagnostic criteria, including but not limited to, lumbar puncture with or without drainage, radiographic parameters (including Evan's index and Frontal-Occipital horn ratio), exclusion of a previously diagnosed neurocognitive disorder, hydrocephalus ex-vacuo, or other forms of communicating and obstructive hydrocephalus [11]. Patients underwent a cognitive and gait analysis before surgery, as is imperative in diagnosing NPH. The diagnosis was unanimously identified by neurology and neurosurgical staff. The study only included patients who also underwent an ETV procedure performed by the first author at either Henry Ford Hospital between July 2007 and April 2009 or Upstate University Hospital between October 2009 and October 2014. All patients had signed and documented informed consent to ETV with cortical biopsy, as met by the institutional surgical consent protocols, and after surgical options, including alternative CSF diversion methods, were discussed. Cortical biopsy was offered to the patient and/or primary caregiver as a diagnostic adjunct. The option of refusal was discussed after the risks were discussed. Patient age, sex, weight, BMI, history of diabetes mellitus, and hypertension were recorded preoperatively. The presence of various risk factors for NPH was also assessed. Patient symptoms were assessed over the course of up to five follow-up appointments. Gait improvement was the primary outcome measure used to determine ETV success. Gait evaluation was performed by subjective assessment of the primary surgeon in conjunction with the patient's primary caregiver. Patients were also assessed for incontinence, dementia symptoms, headaches, memory deterioration, and cognitive function. We defined a positive cortical biopsy result as the presence of β-amyloid or neuritic plaques. The cortical biopsy sample was obtained from the frontal lobe through a burr hole just prior to the insertion of the endoscope into the ventricle using a microdissection tool that was along the path of the endoscope and smaller than the bore of the endoscope. Histological analysis was performed by the institution's designated pathologist, and the characteristics of the sample were described in the pathology report. Patients who did not have the description of amyloid or neuritic plaques were not included in the study.

Independent two-sample t-tests and chi-squared analyses were performed to assess the relationship between ETV success and a variety of continuous and categorical variables, including cortical biopsy results, post-surgical memory disturbance, and post-surgical incontinence. Other analyses were performed to assess the relationships between ETV success and the risk factors studied. Age differences between the groups were assessed for statistical significance using the Mann-Whitney U test. Statistical significance was set at a 95% CI. This retrospective chart analysis was deemed exempt by the institutional review boards of both Henry Ford Hospital and Upstate University Hospital.

## Results

Nineteen patients (53%) had successful ETV at follow-up, compared to 17 (47%) who did not (Table 1).

**Table 1:** Demographics (N=36). ETV: Endoscopic third ventriculostomy.

	ETV Success (N=19)	ETV Failure (N=17)	P-value
Age	65.26 + 9.2	74.58 + 6.8	<0.05
Gender (Female)	9 (47.4%)	8 (47.1%)	>0.05
BMI	27.3 + 4.6	28.0 + 3.4	>0.05
Comorbidity			
Type II Diabetes Mellitus	7 (26.8)	5 (29.4%)	>0.05
Hypertension	10 (52.6%)	7 (41.1%	>0.05
Biopsy Sample (Positive)	4 (21.1%)	6 (35.0%)	>0.05
Follow-up (mo)	8.89 ± 0.39	4.97 ± 0.24	>0.05

Of the 27 patients who underwent both ETV and cortical biopsy, 25 of these patients attended at least one follow-up appointment. There was a difference in the average total follow-up period between the groups (8.9 months in the successful group and 5.0 in the unsuccessful group). The median age of the participants was 72 years. The mean age of patients with successful ETVs was 65.8 ± 6.0 versus 74.5 ± 7.0 for an unsuccessful ETV (p=0.003). Both the treatment and control groups were well-balanced in terms of gender, with each containing 53% male participants. Four of 19 patients with successful ETV had a positive cortical biopsy (21%), compared to six patients with a positive biopsy who had an unsuccessful ETV (35%) (p=0.118) (Table 2).

**Table 2:** Description of patient-related symptoms prior to ETV (N=36) and results of biopsy sampling. ETV: Endoscopic third ventriculostomy.

	Response	N (%)	Positive Biopsy (N=12)	Negative Biopsy (N=15)	P-value
Age			73.6 ± 6.2	69.9 ± 9.6	>0.05
Symptom Duration	<3 months	19 (53%)	7 (58%)	12 (80%)	>0.05
	3 to 6 months	9 (33%)	1 (8%)	8 (53%)	
	>6 months	6 (22%)	1 (8%)	4 (27%)	
Headache	No	6 (22%)	5 (42%)	5 (33%)	>0.05
	Yes	6 (22%)	0 (0%)	1 (7%)	
Memory	No	3 (4%)	2 (17%)	2 (13%)	>0.05
	Yes	8 (30%)	3 (25%)	5 (33%)	
Incontinence	No	6 (22%	4 (33%)	2 (13%)	>0.05
	Yes	8 (30%)	3 (25%)	5 (33%)	
Dementia	No	0	0	0	----
	Yes	1 (4%)	0	1 (7%)	
Combination of Symptoms	Gait only	4 (11%)	3 (25%)	1 (7%)	>0.05
	Gait, Memory	0 (0%)	0	0	
	Gait, Memory, Incontinence	5 (7%)	2 (17%)	3 (20%)	
	Gait, Memory, Dementia	0 (0%)	0	0	
	All four	1 (0%)	0	7%	
Number of symptoms	Less than all four	26	12 (100%)	13 (93%)	>0.05
	All four	1	0	1 (7%)	
Gait improvement	No	11 (41%)	6 (50%)	5 (33%)	>0.05
	Yes	15 (56%)	4(33%)	11 (67%)	

It was found that 67% of the patients with negative biopsies showed gait improvement at the last follow-up appointment, a marker consistent with ETV success (Table 2). Only 33% of patients with positive biopsies showed gait improvement. Ultimately, this study did not find a statistically significant relationship between the presence of amyloid or neuritic plaques on biopsy and ETV success (p=0.188). Memory disturbance and incontinence after surgery were both associated with a lack of gait improvement at the last follow-up appointment (p=0.006 and p=0.0003, respectively). Only one patient had dementia symptoms after ETV to determine whether post-ETV dementia symptoms (as opposed to memory symptoms) were directly correlated with gait improvement.

**Figure 1:**
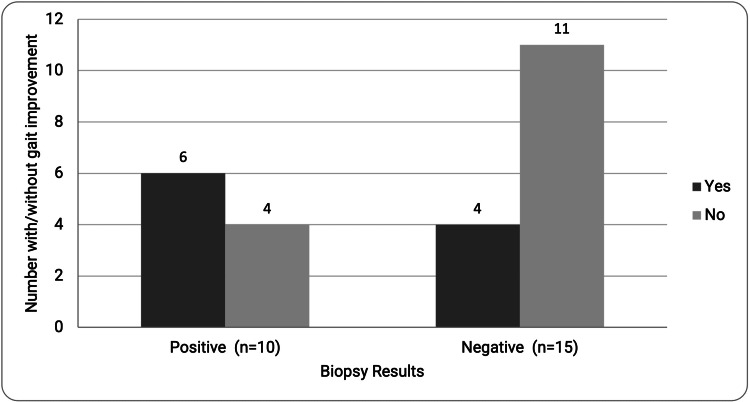
Cortical biopsy results and gait improvement after ETV (N=25 patients). ETV: Endoscopic third ventriculostomy.

Postsurgical headache symptoms did not predict ETV success (p=0.686). To examine the relationship between the duration of postsurgical incontinence, headache, memory, gait, or dementia symptoms and the ultimate ETV outcome, subjects were stratified into groups with symptoms lasting less than 3, 3-6, and >6 months. No relationship was found between any of these groups and ETV success (p=0.369) (Figure 1). Of note, two patients who underwent ETV were found to have ventriculoperitoneal shunt placement within the follow-up period. 

## Discussion

Our study adds to the current literature on the utility of ETV in idiopathic normal-pressure hydrocephalus in patients with evidence of dementia. We described a success rate of 52% in our population, comparable to the current rates of 30-90%. Of the patients who had a positive cortical biopsy, 21% had successful ETV, compared to 35% of patients who had unsuccessful ETV. Although not statistically significant, it is essential to consider that an underlying positive diagnosis of AD may decrease the rate of ETV success in this population. Additionally, younger patients were more likely to experience improved outcomes with ETV. This contrasts with previous studies that found no association between patient age and ETV results.

Several studies have examined a wide variety of possible risk factors to better predict which patients are most likely to benefit from this treatment. These included patient age and sex, etiology of hydrocephalus, surgeon experience, and ETV case volume. ETV success rates have been shown to be lowest in younger patients, which contradicts our findings [9]. Patients at higher risk of developing hydrocephalus, such as post-infectious [10] or post-hemorrhage, are also less likely to have successful ETV [8]. Fukuhara T et al. (n=89) found that patients with a history of intracranial infection were less likely to have success with ETV, while Woodworth GF et al. (n=124) and Dusick JR et al. (n=108) found no such association [12-14]. Amini and Schmidt and Gangemi et al. both found that patients with previous intracranial shunts placed had lower ETV success rates. Gangemi M et al. and Drake JM (n=368) found no association between surgeons' experience with ETV and ETV success rate [15, 16]. These inconsistencies in results warrant further research on this surgical technique for hydrocephalus. 

Another suggested predictor of ETV outcomes in NPH is Alzheimer's pathology. In AD, ventriculomegaly with ex vacuo changes is common. We attempted to study this variable by including cortical biopsies in our investigation, where patients with Alzheimer's are generally found to have β-amyloid extracellular plaques and/or neurofibrillary tangles. Finally, we found that patients with negative cortical biopsies were twice as likely to have successful outcomes with ETV (65%) than those with positive biopsies (35%). This finding was not statistically significant (p=0.188); however, it may be that this study was insufficiently powered to unearth a significant difference. In the future, the examination of a larger population is warranted. In a study examining the biopsy effect in response to shunted hydrocephalus in iNPH with possible dementia, no significant difference was seen in positive or negative biopsy groups [4]. Several studies have previously found that β-amyloid and/or hyperphosphorylated tau pathology negatively impacts CSF diversion, as it has been better studied in the shunt population. For example, Nakajima M found that patients with low (<30 pg/mL) phosphorylated tau levels had better Mini-Mental State Examination (MMSE) results than patients with high (≥30 pg/mL) phosphorylated tau levels. They also found that patients with low phosphorylated Tau had rapid and sustained modified Rankin Scale (mRS) improvement, while patients with high phosphorylated Tau had mRS performance gradually declined [17]. These findings have not just been limited to ETV patients, either. Studies on shunted patients have also shown this relationship. In a study including 145 patients, it was found that patients without amyloid beta and hyperphosphorylated tau pathology in the frontal cortical biopsy and lower BMI had higher health-related quality-of-life outcomes one and five years after shunting [18, 19]. A smaller-scale study of 37 patients found that patients with moderate-to-severe Tau and Aβ pathology had worse results after shunting on cognition and NPH symptom severity scales. Patients with moderate-to-severe pathology had no improvement in gait, cognition, or incontinence at a 4-month follow-up compared to patients with less severe pathology [20].

The strengths of this study include consistency in the surgical technique, geographically diverse patient populations, and a long duration of patient follow-up. However, this study had some limitations. The small sample size is the biggest limitation of this study. There was a difference in the number of patients who had a successful ETV and positive cortical biopsy; however, the difference was not statistically significant. As this was a retrospective study, the authors could not control for any potential biases involved in the selection of the patients for endoscopic third ventriculostomy surgery compared to other interventions, such as ventriculoperitoneal shunt placement. Another limitation is that the surgeries were performed by a single surgeon, albeit in two patient populations. There was also nearly a four-month difference in follow-up time between the successful and unsuccessful groups, limiting the review of subsequent analysis, including those who may have improved thereafter or went on to require additional CSF diversion. This may be due to the shorter follow-up period for unsuccessful patients. However, there was still a nearly 50% decrease in follow-up time compared to the successful ETV group, and the fact that this may have impacted outcome measurements must be considered. Additional radiographic studies were not reviewed, including MRI, which would add a further objective review of ventriculostomy patency. Further comparison studies of patients with iNPH who underwent ETV versus shunt placement and cortical biopsy may provide greater insight into neurodegenerative disease's role.

## Conclusions

In this study, 67% of the patients with negative biopsies had ETV success versus only 33% of the patients with positive biopsies. In contrast, this relationship was not statistically significant. This finding would be worth investigating further with a larger sample size. Older patients with NPH had fewer odds of having a successful ETV. The diagnostic value of a cortical tissue biopsy may help delineate the underlying successes and failures of this intervention for iNPH patients. Further study is needed to elucidate the value of ETV in patients with iNPH and coexisting dementia, as not all patients with iNPH may be candidates for shunt placement.
